# Beyond obstructive azoospermia: emerging insights into reproductive health in men with cystic fibrosis

**DOI:** 10.1007/s10815-026-03896-0

**Published:** 2026-05-18

**Authors:** Andrea Crafa, Aldo E. Calogero, Sarah C. Vij, Rossella Cannarella

**Affiliations:** 1 University of Catania, Catania, Italy; 2 University of Texas at Austin Dell Medical School, Austin, United States

**Keywords:** Cystic fibrosis, CFTR, Male infertility, Nonobstructive azoospermia, Hypogonadism

## Abstract

Cystic fibrosis (CF) is a genetic disorder caused by mutations in the cystic fibrosis transmembrane conductance regulator (CFTR) gene and is classically associated with male infertility due to obstructive azoospermia secondary to congenital bilateral absence of the vas deferens. Increasing evidence suggests that CFTR dysfunction may also contribute to nonobstructive forms of male infertility. Accordingly, CFTR is widely expressed throughout the male reproductive tract, including Sertoli cells, germ cells, and mature spermatozoa. This review summarizes current evidence linking CFTR mutations and variants to hypogonadism, impaired spermatogenesis, altered sperm function, and reduced reproductive outcomes. Clinical studies indicate a high prevalence of testosterone deficiency in men with CF or CBAVD, often occurring in the presence of normal gonadotropin levels. Genetic and meta-analytic data support an association between CFTR variants, particularly the IVS8-5 T polymorphism, and nonobstructive azoospermia. Experimental studies further demonstrate that CFTR plays a critical role in spermatogenesis via regulation of the cAMP-CREB signaling pathway in Sertoli cells and through modulation of microRNA expression affecting germ cell proliferation. CFTR expression in spermatozoa is also implicated in capacitation, motility, and fertilizing capacity through coordinated chloride and bicarbonate transport and interactions with SLC26 family members. Emerging evidence additionally suggests a role for CFTR in early embryonic development, with potential implications for assisted reproductive technology outcomes. Collectively, these findings challenge the traditional view of CF-related male infertility as purely obstructive and highlight CFTR mutations as a potential contributor to nonobstructive infertility. Further studies are required to clarify pathogenic mechanisms and explore targeted therapeutic strategies.

## Introduction

Cystic fibrosis (CF) remains one of the most common genetic disorders, with a global incidence estimated between 1 in 3000 and 1 in 6000 individuals, corresponding to carrier frequencies ranging from 1 in 28 to 1 in 40 [1]. The disease results from mutations in the *cystic fibrosis transmembrane conductance regulator* (CFTR) gene, which encodes a transmembrane protein functioning as a chloride (Cl^–^) and bicarbonate (HCO_3_^−^) ion channel. This protein is directly and indirectly involved in the regulation of ion and water transport across cellular membranes, playing a crucial role in the production of secretions such as mucus [2].

Among the various clinical manifestations of CF, male infertility is a prominent feature. This condition is typically defined as obstructive azoospermia, secondary to congenital bilateral absence of the vas deferens (CBAVD), where different segments of the male reproductive tract, from the epididymal head to the seminal vesicles, are absent [3]. CBAVD is usually characterized by hypoplastic seminal vesicles, low ejaculate volume, and acidic semen (pH < 7), while testicular volume and serum FSH levels remain within normal limits, reflecting an intact hypothalamic–pituitary–gonadal axis. Approximately 98% of men with symptomatic CF present CBAVD upon physical examination [3]. The small minority of fertile CF patients (< 2%) typically carry specific *CFTR* genotypes, such as the 3849 + 10 kb C-T variant [4].

Genetic analyses have shown that between 80 and 97% of CBAVD cases harbor CFTR mutations. Among these, 78% of patients carry at least one mutation, while 53% possess two or more, indicating that even partial impairment of CFTR function significantly contributes to CBAVD pathogenesis [4]. Notably, variants such as the IVS8-5 T polymorphism, the TG repeat tract, and class IV and V mutations are frequently observed in these patients [3]. Several mechanisms have been proposed to explain the link between CFTR dysfunction and CBAVD. One hypothesis suggests that reduced CFTR activity disrupts ion transport within the male genital ducts, leading to increased luminal viscosity, mucus accumulation, and ultimately obstruction of the epididymis and vas deferens. Alternatively, CFTR dysfunction may directly impair Wolffian duct differentiation during embryogenesis, thereby preventing normal development of the male excurrent ducts [4].

The advent of highly effective CFTR modulators (HEMT) has markedly improved clinical outcomes, including pulmonary function, frequency of exacerbations, symptom burden, and body weight. Consequently, life expectancy in CF patients has increased, with mean survival now exceeding 50 years [5]. This improvement has renewed interest in male fertility, as recent evidence suggests that infertility in CF is not exclusively explained by obstructive azoospermia.

From a molecular perspective, CFTR possesses a regulatory (R) domain that is intrinsically disordered, allowing it to participate in multiple protein–protein interactions and regulatory pathways [2]. Furthermore, the cytoplasmic amino (N) and carboxyl (C) termini mediate interactions with a wide range of proteins. The last four residues of the C-terminus form a postsynaptic density 95/discs large/zonula occludens (PDZ)-binding motif, which enables interactions with PDZ-domain proteins, extending CFTR’s functional role beyond Cl^−^ and HCO_3_^−^ transport [6].

The relevance of CFTR to male fertility is supported by its widespread expression throughout the male reproductive tract. In the seminiferous tubules, CFTR is expressed in Sertoli cells and all germ cell populations, with notable enrichment in primary spermatocytes and elongating spermatids. Within Sertoli cells, CFTR contributes to pathways leading to CREB phosphorylation and the transcription of spermatogenic genes; mediates bicarbonate flux for pH regulation; and supports tight junction formation, which is critical for blood–testis barrier integrity and germ cell development [2]. Moreover, CFTR exhibits stage-specific expression in pachytene spermatocytes and elongated spermatids, suggesting a role in spermiogenesis, and is also localized to the equatorial segment of mature spermatozoa, implicating it in sperm motility [2].

Consequently, CFTR dysfunction may compromise fertility via multiple mechanisms, including altered luminal fluid composition, impaired epithelial differentiation, and defective sperm motility or capacitation. These processes may further be mediated by disrupted interactions between CFTR and proteins essential for male fertility, such as aquaporins (AQPs), epithelial sodium channel (ENaC), solute carrier family (SLC16) transporters, β-catenin, soluble NSF attachment protein receptor (SNARE) proteins, lysophosphatidic acid receptor 2 (LPAR)2, and zonula occludens-1 (ZO-1). Whereas AQPs, ENaC, and SLC16 transporters regulate extracellular fluid composition, β-catenin, SNARE proteins, LPAR2, and ZO-1 are pivotal for epithelial development and differentiation [2].

This review aims to synthesize current knowledge on the role of CFTR in the pathogenesis of nonobstructive male infertility, emphasizing the importance of further research into minor CFTR mutations and their potential diagnostic and therapeutic implications.

## CFTR mutations and hypogonadism

Several studies have documented a high prevalence of hypogonadism in men with CF or CBAVD. An analysis of the TriNetX database, including 14,033 men over 18 years of age with CF and 3439 men with CBAVD, revealed that only 10.1% of CF patients and 8.9% of CBAVD patients underwent evaluation of serum testosterone levels. Among those tested, 32.7% of CF patients and 43% of CBAVD patients were found to have low testosterone concentrations, underscoring both the clinical relevance of this issue and the limited frequency with which it is investigated [7].

In another recent study, 41.7% of 12 CF patients exhibited elevated FSH levels (> 7.6 IU/L), while 16.7% were diagnosed with apparently unexplained hypogonadism, findings that point toward possible testicular dysfunction in this population [8]. Consistent with these data, a separate investigation involving 71 individuals with biallelic *CFTR* mutations reported that 22% had reduced testosterone levels despite normal gonadotropin concentrations [9]. Similarly, another study of 69 men with CF and mean age of 33.34 ± 10.98 years found that 27.54% of participants had value of testosterone below 300 ng/dL [10].

The pathophysiological mechanisms underlying testosterone deficiency in CF remain incompletely understood. Contributing factors may include recurrent infections, chronic systemic inflammation, malnutrition, and long-term glucocorticoid therapy [11, 12], all of which are known to disrupt either the activity of GnRH-secreting neurons or steroidogenesis in Leydig cells. Additionally, emerging evidence suggests a direct involvement of mutated CFTR in the impairment of neuropeptide vesicle trafficking within GnRH-secreting neurons [13], potentially explaining why most studies report reduced testosterone levels in the presence of normal gonadotropin profiles.

## CFTR mutations and nonobstructive azoospermia (NOA)

The hypothesis that spermatogenesis may be compromised in patients harboring *CFTR* gene mutations has long been recognized in the literature. As early as 1996, van der Ven and colleagues investigated 13 *CFTR* mutations in 127 unrelated men presenting with various forms of impaired sperm quality. They reported that 17.5% of otherwise healthy men with infertility due to reduced sperm quality, and 14.3% of men with azoospermia, carried at least one *CFTR* mutation. This frequency was significantly higher than the expected carrier rate in the local population (*P* = 0.00139). Notably, no mutations were identified in a control cohort of 26 men with normal semen parameters. The most prevalent variant was G542X (8/18; 44%), followed by ΔF508 (7/18; 33%). Four additional mutations (G551D, R117H, 621 + 1G → T, and 3849 + 10kbC → T) were each detected in a single case. These findings suggested that CFTR may influence spermatogenesis or sperm maturation, independently of its established role in the development of the epididymal glands and the vas deferens [14].

Subsequent studies have confirmed the contribution of *CFTR* mutations to the pathogenesis of spermatogenic defects [15–17]. Among the most extensively investigated variants is the 5 T polymorphism in the noncoding IVS8 region of the *CFTR* gene. This polymorphism represents a variant of the IVS8-(*n*)T tract, where *n* denotes the number of thymidine residues, with allelic variants containing 5, 7, or 9 T residues. The 7 T and 9 T alleles are generally considered functionally neutral, whereas the 5 T variant is associated with aberrant splicing of exon 9, resulting in CFTR transcripts lacking this exon and yielding a dysfunctional protein [18]. The likelihood of exon 9 skipping is further modulated by the number of TG repeats upstream of the IVS8-5 T allele. While the typical number is 11, the presence of 12 or 13 TG repeats in cis with a 5 T allele exacerbates the impairment of CFTR protein synthesis, thereby contributing to male infertility [19].

A recent meta-analysis synthesized available evidence regarding these variants and spermatogenesis. The study included 20 investigations encompassing 6423 patients and 5834 controls. The analysis demonstrated that the IVS8-5 T allele was associated with an approximately threefold increased risk of impaired spermatogenesis (OR = 2.84). Stratified analyses revealed a stronger effect in non-European populations (OR = 4.50) compared with European cohorts (OR = 1.28). Furthermore, the risk was higher in studies including both CBAVD and nonobstructive azoospermia (NOA) patients (OR = 3.18) than in those restricted to NOA (OR = 1.82). The authors concluded that the IVS8-5 T variant contributes to defective spermatogenesis, although additional studies are required to substantiate this hypothesis [18]. Similar findings were reported by another meta-analysis of 14 studies, which demonstrated a positive association between the IVS8-5 T mutation and the risk of nonobstructive male infertility (OR = 1.69; 95% CI 1.12–2.55). Notably, this association was even stronger when the analysis was restricted to populations with nonobstructive azoospermia (OR = 2.62; 95% CI 1.49–4.61) [20].

In a separate investigation, a Chinese study identified a loss-of-function *CFTR* p.G970D missense mutation in a patient with concurrent CBAVD and NOA. Retrospective analysis of 122 Chinese CBAVD patients revealed this mutation in 4.1% of cases. Functional assessment was performed using murine testis-derived cell lines engineered with the homologous Cftr p.G965D mutation. This variant proved lethal in spermatogonial stem cells (C18-4) and spermatogonia (GC-1spg), and impaired proliferation of spermatocytes [GC-2(spd)ts] and Sertoli cells (TM4). In GC-2(spd)ts cells harboring the mutation, splicing abnormalities and reduced CFTR expression were observed, which likely contribute to the spermatogenic phenotype.

Collectively, these findings suggest that CFTR p.G970D is a pathogenic mutation associated with CBAVD and impaired spermatogenesis in the Chinese population [21].

A mechanistic study has further implicated CFTR in the regulation of spermatogenesis through the cAMP–CREB signaling cascade in Sertoli cells. In CFTR knockout mice (S489X), testis size and weight, daily sperm production, and epididymal sperm counts were significantly reduced compared with wild-type controls. Reduced expression of Protamine-2, a marker of spermatids and mature spermatozoa, was also observed. Immunofluorescence demonstrated reduced expression of cAMP-response element binding protein (CREB) in Sertoli cells of homozygous mutants. CFTR-mediated bicarbonate influx into Sertoli cells is proposed to regulate FSH-induced activation of the cAMP–CREB pathway via soluble adenylyl cyclase (sAC). Consistent with this model, pharmacological inhibition with CFTRinh172 and KH7 abrogated bicarbonate-induced enhancement of FSH-stimulated cAMP production. These findings were corroborated also in humans where testicular samples from NOA patients exhibited reduced expression of CFTR, CREB, and Protamine-2 compared to controls, suggesting that disruption of the CFTR–CREB axis may underlie azoospermia [22].

Additional evidence suggests that defective CFTR may dysregulate spermatogenesis via altered microRNA expression. Specifically, dysfunctional CFTR has been linked to upregulation of microRNA-15b (miR-15b), which represses CDC25A, a cell cycle regulator, thereby inhibiting germ cell proliferation. Overexpression of miR-15b appears to result from dysregulated interactions between Ago2 and Dicer1 determined by the mutated CFTR, leading to enhanced Dicer1 activity and increased miR-15b levels [23].

Despite compelling evidence, some studies have failed to demonstrate an association between *CFTR* mutations and spermatogenesis defects [24–27]. For instance, a case–control study involving 100 fertile men and 100 NOA patients screened for F508del and I507del mutations found no significant correlation [24]. Similarly, an investigation of 45 NOA patients did not identify associations between *CFTR* mutations or the IVS8-5 T variant and primary spermatogenic dysfunction [25].

Taken together, these findings underscore the need for further investigations to clarify which *CFTR* mutations or variants are most strongly associated with spermatogenic impairment and to elucidate their pathogenic mechanisms.

## CFTR protein in sperm function and fertilization

In addition to their established role in the pathogenesis of secretory azoospermia and spermatogenic impairment, mutations in the *CFTR* gene are also thought to contribute to alterations in sperm quality. This hypothesis is supported by evidence demonstrating CFTR protein expression in the equatorial region of spermatozoa in both humans and animal models [28]. An in vitro investigation on human spermatozoa reported that CFTR expression in this region is reduced in infertile patients with normozoospermia as well as in individuals with abnormal semen parameters, thereby reinforcing the involvement of CFTR in sperm quality [29]. Consistently, a study of 282 healthy fertile men with normal semen parameters found a mild but statistically significant positive correlation between CFTR expression and sperm progressive motility (*r* = 0.221) as well as normal morphology (*r* = 0.202), whereas no association was detected with semen volume, sperm concentration, or total sperm count [30]. Furthermore, in a cohort of 201 healthy fertile men stratified into three age groups (20–40 years, *n* = 64; 40–60 years, *n* = 61; > 60 years, *n* = 76), an age-dependent decline in CFTR expression at the equatorial segment and neck region of spermatozoa was observed, which correlated with reduced forward motility and decreased HCO_3_⁻ sensitivity, both essential for sperm capacitation [31].

Additional evidence for the role of CFTR in sperm quality comes from studies in uremic patients, in whom CFTR protein levels are markedly lower compared to both fertile and infertile controls; notably, renal transplantation partially restored CFTR expression, concomitant with improvements in semen parameters. These findings suggest that sperm CFTR expression is associated with the fertility decline observed in uremic patients and may serve as a potential biomarker for assessing reproductive potential in this population [32].

The localization of CFTR in the equatorial region further implies its participation in capacitation and, consequently, in fertilization competence. Supporting this view, in vitro experiments demonstrated that exposure of spermatozoa to CFTR inhibitors impairs progesterone-induced capacitation, suppresses hyperactivated motility and acrosomal activity, and ultimately diminishes the ability of sperm to penetrate the oocyte [27]. Both chloride and bicarbonate anions appear to be critical in these processes. Indeed, multiple in vitro studies in humans and animals have sought to clarify the mechanisms by which CFTR contributes to capacitation. At the molecular level, one of the earliest capacitation events is the HCO_3_⁻-dependent activation of sAC, leading to cAMP synthesis and subsequent activation of PKA [33]. Importantly, bicarbonate influx itself appears to be modulated by chloride levels. Indeed, sperm incubated under progressively lower chloride concentrations exhibit impaired capacitation, even in the presence of adequate bicarbonate, as evidenced by reduced intracellular alkalinization, inhibition of sAC activity, and diminished capacitation-associated protein tyrosine phosphorylation. This indicates a requirement for chloride in mediating bicarbonate entry into spermatozoa [34, 35].

Although CFTR is a major chloride transporter in sperm, it is not the sole pathway; its deficiency can potentially be compensated by alternative channels such as Cl⁻-selective ion channels, GABAA receptor/Cl⁻ channels, Ca^2^⁺-activated Cl⁻ channels, and Na⁺/K⁺/Cl⁻ cotransporters [34]. Nevertheless, in vitro studies using various chloride transport inhibitors [CFTRinh-172, adjudin, diphenylamine-2-carboxylate (DPC), 4′,4′-diisothiocyanostilbene-2′,2-disulfonic acid (DIDS), and bicuculline] demonstrated differential inhibitory effects on sperm capacitation, with the strongest inhibition induced by CFTRinh-172, a high-affinity CFTR blocker, and the weakest by bicuculline, a GABAA receptor/Cl⁻ channel inhibitor [36]. These results indicate the pivotal role of CFTR in chloride transport and confirm its central involvement in capacitation-related processes [27].

Although CFTR can independently mediate chloride/bicarbonate exchange [28], current evidence suggests that it functions within multimolecular complexes involving other transporters. In particular, SLC26A3 has been implicated [34]. Experimental inhibition of CFTR with CFTRinh-172, of SLC26A3 with niflumic acid, or blockade with SLC26A3-specific antibodies all impaired sperm capacitation, supporting this interaction [34]. Similarly, an in vitro study demonstrated a functional interplay between CFTR and another member of the SLC26 family, SLC26A8 (encoded by TAT1). Tat1 knockout mice lacked sperm capacitation, pointing towards the essential role of the CFTR-SLC26A8 interaction [37]. Consistent with these findings, missense mutations in SLC26A8 have been identified in men with asthenozoospermia, likely disrupting CFTR-SLC26A8 complex formation and impairing the associated regulatory mechanisms [38]. In parallel, a heterozygous mutation (c.2062 G > C; p.Asp688His) in SLC26A3 was detected in 3.2% of a cohort of 283 infertile men, where it was proposed to interfere with CFTR-SLC26A3 interactions and consequently compromise chloride/bicarbonate homeostasis in spermatozoa [39].

## CFTR in embryonic development: potential implications for ART outcomes

Among the multiple functions attributed to CFTR in the context of fertility, evidence also suggests a contribution to embryonic development. An in vitro study on porcine oviductal cells demonstrated that CFTR is involved in the elevation of bicarbonate ion concentrations within the tubal microenvironment. In turn, bicarbonate, through activation of the sAC signaling pathway, was shown to promote the progression of 2-cell embryos to the morula or blastocyst stage [40]. In this process, not only CFTR but also members of the SLC26 family, particularly isoforms A3 and A6, appear to play a role, as indicated by the inhibition of embryonic growth following the use of specific inhibitors targeting these transporters [41]. Similarly, an in vitro study on mouse embryonic stem cells (mESCs) revealed that CFTR exerts a pivotal role in embryogenesis, particularly influencing mesoendodermal development. This was supported by the observation of reduced expression of mesodermal and endodermal differentiation markers in mESCs derived from CFTR knockout mice compared with wild-type (WT) counterparts [42]. From a mechanistic perspective, given the well-established involvement of the Wnt/β-catenin pathway in mesoendodermal differentiation, the authors investigated β-catenin expression levels. A significant reduction in β-catenin and its downstream target genes was detected in mESCs from CFTR knockout mice compared to WT controls. Furthermore, co-immunoprecipitation assays confirmed a physical interaction between CFTR and β-catenin, suggesting that CFTR mutations at the embryonic level may impair development through dysregulation of the Wnt/β-catenin signaling cascade [42].

Considering the role of CFTR in both sperm function and embryonic development, several studies have hypothesized its potential involvement in natural fertilization as well as in the outcomes of assisted reproductive techniques (ARTs). In this regard, a study conducted on 135 healthy couples, stratified according to fertility status, reported significantly reduced CFTR expression in spermatozoa from normozoospermic males belonging to couples with infertility compared to fertile controls. Receiver operating characteristic (ROC) analysis identified a threshold value of 43.75% *CFTR* expression, below which fertility was impaired. Notably, patients with expression levels under this cut-off exhibited a significantly lower cumulative pregnancy rate compared with those exceeding the threshold (49.3% vs. 80.6%) [43].

With regard to ART outcomes, an analysis of 1014 intracytoplasmic sperm injection (ICSI) cycles, categorized by the presence of CBAVD versus non-CBAVD obstruction, demonstrated higher miscarriage and stillbirth rates per embryo transferred in men with CBAVD compared to those with non-CBAVD obstruction. Conversely, the live birth rate per embryo transferred was lower in the CBAVD group. Given the significantly higher frequency of CFTR mutations among CBAVD patients, the authors further assessed reproductive outcomes based on mutational status. The results indicated increased miscarriage and stillbirth rates per embryo transferred in carriers of CFTR mutations compared with noncarriers. Moreover, the prevalence of CFTR mutations was greater in patients experiencing miscarriages or stillbirths than in those achieving live births, supporting the hypothesis that CFTR dysfunction contributes to reduced reproductive success in CBAVD patients. These findings point to diminished reproductive potential of spermatozoa from individuals harboring CFTR mutations and suggest a possible role of paternal CFTR transmission in embryonic development [44].

In another study, the comparison between 10 patients with CF and 29 patients with CBAVD, all carrying *CFTR* mutations, showed that CF patients exhibited lower surgical sperm retrieval and fertilization rates following ICSI compared with the CBAVD group. This further supports the impact of CFTR dysfunction on spermatogenesis and indicates that the severity of CFTR impairment influences fertility outcomes [45]. Nevertheless, additional studies are required to clarify the relative contribution of impaired sperm quality versus direct effects of paternal CFTR transmission on embryonic development.

## Future directions and conclusions

**Fig. 1:**
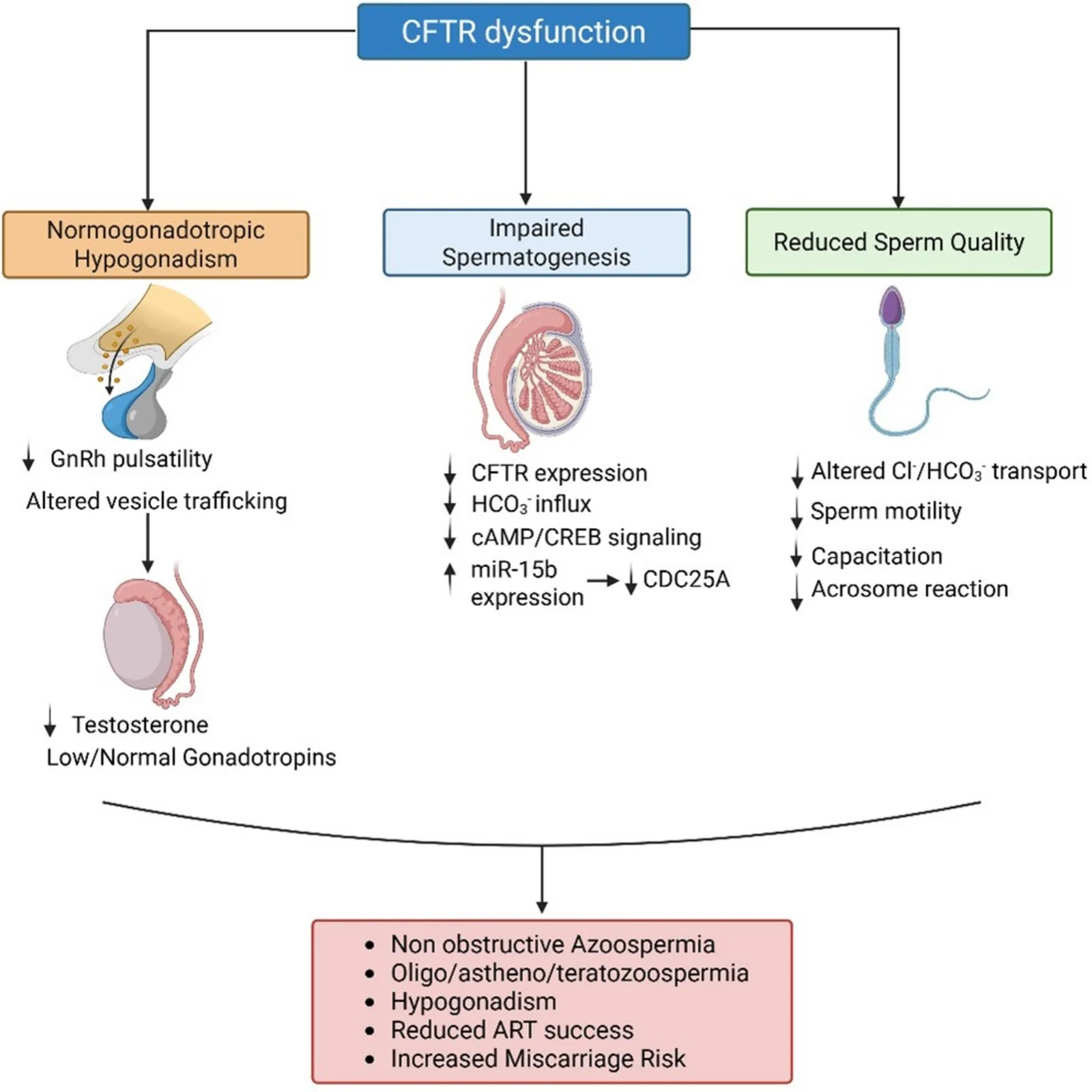
Cystic fibrosis transmembrane conductance regulator (CFTR) dysfunction contributes to nonobstructive male infertility through combined effects on the hypothalamic–pituitary–gonadal axis, Sertoli cell signaling, spermatogenesis, and sperm capacitation. These mechanisms collectively result in hypogonadism, impaired sperm quality, and reduced reproductive potential

The evidence discussed thus far indicates that the classical paradigm linking CFTR mutations exclusively to obstructive azoospermia has been partially revised (Fig. 1). Genetic analysis of the *CFTR* gene should therefore also be considered in patients presenting with secretory forms of azoospermia and/or a history of infertility associated with unexplained alterations in sperm parameters. However, the precise mechanisms through which CFTR mutations may affect spermatogenesis and embryonic development remain incompletely understood. Similarly, the impact of these mutations on the outcomes of ARTs is yet to be fully elucidated. Most of the studies conducted to date are retrospective in nature and lack appropriate adjustment for key confounding variables, including hormonal status, testicular volume, and the presence of comorbidities that may influence sperm quality and/or treatment success. Current evidence nonetheless highlights new perspectives regarding the potential use of CFTR-targeted therapeutic approaches aimed at improving sperm parameters or ART success rates in mutation carriers. To date, however, no studies have directly addressed this possibility. In conclusion, although additional research is necessary to clarify the role and underlying mechanisms of *CFTR* mutations in nonobstructive infertility, such mutations could constitute a treatable cause of male infertility, highlighting the need for dedicated studies on targeted therapeutic approaches.

## Data Availability

Not applicable.
